# Cutaneous *Mycobacterium haemophilum* infections in immunocompromised patients in a tertiary hospital in Bangkok, Thailand: under-reported/under-recognized infection

**DOI:** 10.1099/jmmcr.0.002618

**Published:** 2014-12-01

**Authors:** Thitirat Tangkosakul, Poonpilas Hongmanee, Kumthorn Malathum

**Affiliations:** ^1^​ Department of Medicine, Lerdsin Hospital, Bangkok, Thailand; ^2^​ Department of Pathology, Ramathibodi Hospital, Mahidol University, Bangkok, Thailand; ^3^​ Faculty of Medicine, Ramathibodi Hospital, Mahidol University, Bangkok, Thailand

**Keywords:** cutaneous infection, iron supplement culture, *M. haemophilum*

## Abstract

**Introduction:**

*Mycobacterium haemophilum* is one of the non-tuberculous mycobacteria (NTM) that can cause cutaneous infection. As acid-fast staining cannot distinguish NTM from *Mycobacterium tuberculosis*, and as skin culture for *M. haemophilum* is not performed routinely, the diagnosis of *M. haemophilum* infection in Thailand is rarely made.

**Case presentation:**

Between 2006 and 2009, five patients with *M. haemophilum* infection were diagnosed in Ramathibodi Hospital, a tertiary care centre in Bangkok, Thailand. The patients were aged 3, 29, 47, 75 and 76 years, and four were immunocompromised. Three patients received immunosuppressive medication. Most patients presented with subacute cutaneous infection. A suboptimal response to conventional antibiotics raised suspicions of *M. haemophilum* cutaneous infections, which can occur in immunocompromised patients. Diagnoses of these cases were made by skin culture for mycobacteria at an incubating temperature of around 30 °C with iron supplementation, DNA sequencing, or PCR/restriction enzyme analysis. Rifampicin, ofloxacin and clarithromycin were active against all isolates, whereas ethambutol and streptomycin were inactive.

**Conclusion:**

Skin culture should be performed under special conditions or molecular technique should be used to identify *M. haemophilum* in susceptible patients.

## Introduction

*Mycobacterium haemophilum* is a fastidious organism that has rarely been documented as a cause of human infection. The epidemiology of *M. haemophilum* infection is largely unknown, as are its reservoir and its mode of transmission. An increasing number of infections with this pathogen have been reported, particularly in immunocompromised patients. Recognized clinical syndromes include pulmonary disease, lymphadenitis, catheter-related infections, rheumatological disease and cutaneous infections (White *et al*., 1999). *M. haemophilum* was first isolated in 1978 from ulcerating skin lesions in a patient with Hodgkin’s disease (Sompolinsky *et al*., 1979). Recently, a published report from Taiwan and Lebanon described skin and soft-tissue infections caused by non-tuberculous mycobacteria (NTM) (Liao *et al*., 2007). The most common species was *Mycobacterium marinum*. Other species included *Mycobacterium avium* complex, *Mycobacterium fortuitum*, *Mycobacterium kansasii* and some unspecified species. However, *M. haemophilum* was not specifically mentioned. In contrast, in developed countries, *M. avium* complex was common while rapidly growing mycobacteria (RGM) was found less than in Asia. To date, there are only a few reports of *M. haemophilum* infections from Thailand. The first was a series of patients who were not infected with human immunodeficiency virus (HIV) but had disseminated NTM infection associated with Sweet’s syndrome. A patient in this series had *M. haemophilum* infection but the specific site of infection was not mentioned (Chetchotisakd *et al*., 2007). The second report was a British patient at King Chulalongkorn Memorial Hospital, Bangkok, Thailand, who had AIDS and a brain abscess due to *Mycobacterium simiae* and *M. haemophilum* infection (Phowthongkum *et al*., 2008). In the current study, the authors gathered data between 2006 and 2009 from five patients with only *M. haemophilum* infections who were diagnosed in Ramathibodi Hospital, a medical school and tertiary care centre in Bangkok, Thailand.

We retrospectively retrieved data for patients who were diagnosed with *M. haemophilum* infection at our institution between 2006 and 2009. The diagnoses of all cases were established based on acid-fast stained and culture in a BACTEC MGIT 960 system (Becton Dickinson) or on Lowenstein–Jensen medium (BBL, Becton Dickinson). In cases where *M. haemophilum* infection was suspected, special conditions, such as incubation at 30 °C with iron supplementation, were used. The organism was identified on the basis of 16S rRNA gene sequence analysis using an automated sequencing ABI Prism 310 Genetic Analyzer. The first case was also identified using PCR/restriction endonuclease analysis (PCR-REA) of the genes encoding the 65 kDa heat-shock protein (*hsp65*) and RNA polymerase β-subunit (*rpoB*), which was performed in another hospital by (Cheunoy *et al*., 2005). Drug susceptibility test was determined by broth microdilution supplemented with haemin. Data regarding gender, age, duration of disease, site of lesions and treatment modalities were collected.

## Cases series

**Fig. 1.**
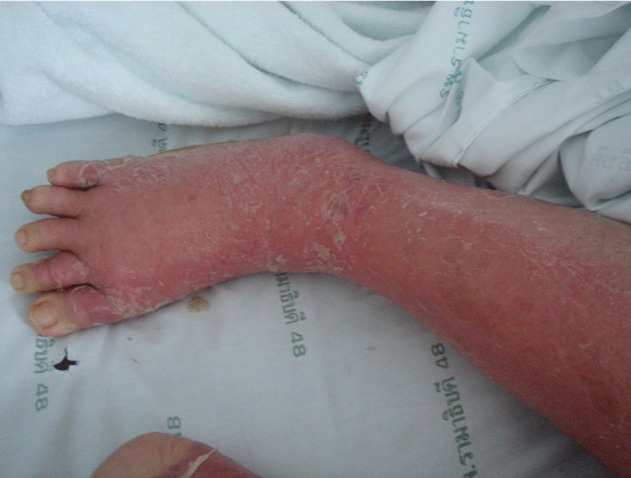
Extensive cellulitis of the legs of one of the patients.

Between 2006 and 2009, five patients with *M. haemophilum* infection were diagnosed in Ramathibodi Hospital, a medical school and tertiary care centre in Bangkok, Thailand (Table 1). The patients were aged 3, 29, 47, 75 and 76 years and presented with subacute to chronic cutaneous infections on the extremities, which were not improved after treatment with conventional antibiotics. Four were immunocompromised patients; one had HIV infection with a CD4^+^ count of 25 cells mm^−3^ and three were taking immunosuppressive medications for systemic lupus erythematosus, rheumatoid arthritis or myasthenia gravis. The first patient (case 1) had multiple discrete, ill-defined erythematous, warm, tender and indurated plaques as well as fluctuating nodules in the subcutaneous tissue of both arms and both legs, mimicking cellulitis of the arms and legs (Fig. 1). The cutaneous lesions of the second and third patients (cases 2 and 3) were singular, small violaceous papules that later became tender and were located on extremities, indicating a preference for lower-than-normal body temperature growth requirements. Case 3 had an HIV infection, which was finally diagnosed as central nervous system (CNS) lymphoma with a skin lesion that was found incidentally on her upper extremities. The youngest patient (case 4) was a 3-year-old previously healthy boy who presented with cervical lymphadenitis for 1 month. Acid-fast staining of the specimens was positive in the first, second and fifth cases. All specimens were positive for acid-fast bacelli from MGIT cultures but failed to grow in conventional mycobacterial culture and were therefore processed by incubating at 30 °C with iron supplementation. Only the first patient had a positive blood culture, and PCR-REA of the *hsp65* and *rpoB* genes confirmed the diagnosis. The species identification of the organisms from all specimens was confirmed by DNA sequencing. Drug susceptibility testing was determined only for the isolates obtained from the second, third and fourth cases by the broth microdilution method supplemented with haemin (Bernard *et al*., 1993). This method without haemin has been showed to correlate well with the agar disk elution method (NCCLS, 2003) when tested against *M. kansasii*. The results showed that rifampicin, ofloxacin and clarithromycin appeared to be active against all isolates with MICs of ≤0.5, 1.0 and ≤1.0 µg ml^−1^, respectively, whereas ethambutol and streptomycin were inactive with MICs of >16 µg ml^−1^. The first patient was treated with azithromycin, ciprofloxacin and rifampicin. Her skin lesions improved and she recovered completely after 9 months of treatment. The second patient’s lesion improved after clarithromycin, ciprofloxacin and rifampicin were prescribed instead of the standard anti-tuberculous drugs. The lesions gradually improved with post-inflammatory hyperpigmentation of skin. She continued treatment for 1 year with no evidence of residual disease or relapse. The patient with the HIV infection (case 3) died from CNS lymphoma before definite treatment. Case 4 was treated with one active drug, rifampicin. Case 5 received one active drug, ofloxacin, but immunosuppressive drugs were also decreased and the lesion still improved.

## Discussion

In this series of five patients, four were immunocompromised, suggesting an association between immunosuppression and infections with NTM. However, we also had a healthy 3-year-old boy who presented with lymphadenitis, as has been reported previously (Lindeboom *et al*., 2005). Typical lesions caused by *M. haemophilum* are usually singular, small violaceous papules that later become tender and suppurative, culminating in painful draining ulcers. Less frequently, they present as cysts, scaly plaques or focal panniculitis (White *et al*., 1999). Multiple ascending lesions resembling sporotrichosis may occur. Septic arthritis and osteomyelitis are less common manifestations and when they do occur, skin disease may also be present.

Laboratory diagnosis of *M. haemophilum* requires special conditions, i.e. an incubating temperature around 30–32 °C, rather than 37 °C. In addition, it grows on egg- or agar-based medium only if supplemented with haemin or ferric ammonium citrate. PCR-REA was carried out by amplification of the *hsp65* and *rpoB* genes. The results showed concordant percentages of 100, 98.8 and 83.3 %, respectively, for *M. tuberculosis* and rapidly and slowly growing mycobacteria, compared with biochemical identification. This method is not considered to be sufficiently sensitive for direct testing on clinical specimens except when the mycobacterial load in the sample is sufficiently high (Da Mata *et al*., 2008). A line-probe assay such as INNO-LiPA and GenoType are alternative techniques. Importantly, gene sequencing of the 16S rRNA gene is useful for rapid identification of *Mycobacterium* spp. by comparing the obtained sequences with data in GenBank.

Most strains of *M. haemophilum* demonstrate *in vitro* susceptibility to ciprofloxacin, clarithromycin, rifamycins and clofazimine. Isolates are usually resistant to isoniazid, ethambutol and pyrazinamide, while susceptibility to doxycycline, minocycline, *para*-aminosalicylic acid and amikacin is variable (Elsayed & Read, 2006). There are currently no standard guidelines on antibiotic treatment for *M. haemophilum* infection and no standardized antimicrobial susceptibility tests, although a recent Clinical and Laboratory Standards Institute document includes recommendations for an agar disk elution method for *M. haemophilum* (CLSI, 2011). Furthermore, it is not clear how these *in vitro* test results predict the clinical response. Consequently, the duration of therapy is not well defined and depends on the therapeutic response and the magnitude of immunodeficiency; for example, improvement of cutaneous *M. haemophilum* infection in an AIDS patient can be seen only after receiving antiretroviral drugs for HIV infection and recovering CD4^+^ cell counts (Paech *et al.*, 2002).

In summary, *M. haemophilum* infections should be included in the differential diagnosis of such a presentation as well as lymphadenopathy, and the microbiology laboratory personnel must be informed when this organism is suspected so that appropriate culture procedures can be employed.

**Table 1.** Demographic data of the five patients in this study: 2006–2009

Case no.	Sex/age	Duration of illness (weeks)	Underlying disease	Current medication	Treatment*	Outcome
1	F/75	3	Rheumatoid arthritis	Prednisolone 5 mg/day	Azithro, Cpfx, Rif	Cured
2	F/76	2	Myasthenia gravis; type 2 diabetes mellitus	Prednisolone 5 mg every other day; azathioprine 100 mg/day	Clr, Cpfx, Rif	Cured
3	F/29	4	AIDS, CD4^+^ 25 cells/mm^−3^ (2 %), primary CNS lymphoma	None	None	Died of lymphoma
4	M/3	4	None	None	Anti-tuberculous drug; susceptible only to rifampicin	Cured
5	F/47	1	Systemic lupus erythematosus	Prednisolone 20 mg/day; azathioprine 50 mg/day†	Anti-tuberculous drug; susceptible only to ofloxacin	Cured

* Azithro, azithromycin; Clr, clarithromycin; Cpfx, ciprofloxacin; Rif, rifampicin.

† Azathioprine was discontinued and the dose of prednisolone was reduced to 10 mg/day.

## Abbreviations

CNS: central nervous system
HIV: human immunodeficiency virus
NTM: non-tuberculous mycobacteria
REA: restriction endonuclease analysis.
